# Supplementary material for: Computer-based intervention for residents of domestic violence shelters with substance use: A randomized pilot study
Source: PLoS One. 2023 May 25;18(5):e0285560. doi: 10.1371/journal.pone.0285560 (PMC10212146; doi:10.1371/journal.pone.0285560)
Supplement: S1 File — (DOCX) [file pone.0285560.s001.docx]

**Supplemental file 1: Summary of unadjusted outcomes at baseline, 3 and 6 months by study condition**

| **Outcome** | **Time point** | **Control Median (Q1-Q3)** | **Computerized intervention**  **Median (Q1-Q3)** | **P-value** |
| --- | --- | --- | --- | --- |
| Composite Abuse Scale – Victimization (lower is better) | Baseline | 74 (41-110.5) | 51 (38-63) | N/A |
|  | 3 months | 2 (0-17) | 3 (0-17) | .62 |
|  | 6 months | 0 (0-6) | 7 (0-24) | .02 |
| Composite Abuse Scale – Perpetration (lower is better) | Baseline | 5.5 (0-20) | 10 (8-18) | N/A |
|  | 3 months | 0 (0-2) | 1 (0-7) | .15 |
|  | 6 months | 0 (0-2) | 2 (0-4) | .03 |

| Cyber-stalking Scale score (lower is better) | Baseline | 10 (4-12) | 10 (6-12) | N/A |
| --- | --- | --- | --- | --- |
|  | 3 months | 0 (0-6) | 6 (0-8) | .20 |
|  | 6 months | 0 (0-6) | 3 (0-8) | .14 |
| Safety Behavior Checklist total score (higher is better) | Baseline | 8 (5-11_ | 7 (3-9) | N/A |
|  | 3 months | 6 (1-11) | 6 (0-7) | .52 |
|  | 6 months | 3 (0-9) | 2 (0-5) | .34 |
| TLFB heavy drinking/drug using days (lower is better) | Baseline | 61 (21-92) | 50 (19-86) | N/A |
|  | 3 months | 26 (1-64) | 11 (1-64) | .61 |
|  | 6 months | 15 (1-84) | 20 (2-79) | .89 |
| TSR number of substance use treatment times (higher is better) | Baseline | 1 (0-12) | 0 (0-1) | N/A |
|  | 3 months | 0 (0-3) | 0 (0-3.5) | .66 |
|  | 6 months | 0 (0-1) | 0 (0-1) | .99 |
|  |  | **Mean (SD)** | **Mean (SD)** | **p** |
| Self-efficacy to reduce substance use (higher is better) | Baseline | 4.58 (1.32) | 4.36 (1.11) | N/A |
|  | 3 months | 4.69 (0.88) | 3.83 (1.20) | .01 |
|  | 6 months | 4.13 (1.32) | 3.95 (1.28) | .65 |
| Readiness to reduce substance use (higher is better) | Baseline | 3.67 (1.76) | 3.96 (1.54) | N/A |
|  | 3 months | 3.13 (1.96) | 3.89 (1.53) | .18 |
|  | 6 months | 3.26 (1.96) | 3.62 (1.86) | .54 |

*Self-efficacy and readiness were compared used t-tests
