# Supplementary material for: Computer-based intervention for residents of domestic violence shelters with substance use: A randomized pilot study
Source: PLoS One. 2023 May 25;18(5):e0285560. doi: 10.1371/journal.pone.0285560 (PMC10212146; doi:10.1371/journal.pone.0285560)
Supplement: S2 File — (DOCX) [file pone.0285560.s002.docx]

**Computer-based intervention for battered sheltered women with substance use**

**Overview**

Substance use and intimate partner violence victimization (IPV) are significant and interrelated public health problems facing women. For battered women, the presence of substance use increases the risk of re-victimization and the risk of more severe abuse. Moreover, given the multitude of competing concerns and the high degree of stress faced by battered women during and after their shelter stay, women with prior substance use problems are at high risk for substance use relapse. Further, substance use difficulties can compromise battered women’s ability to access and effectively use integral community resources, interfering with their ability to establish long-term safety for themselves and their children.

Battered women’s shelters provide emergency shelter to approximately 300,000 women and children each year. A prime time to intervene with battered women might be when they enter a shelter and have already initiated a change in their lives. Thus, a shelter-based intervention for battered women that addresses substance use problems might reduce the risk of substance use, reduce the risk of future IPV, and improve utilization of substance use treatment. Recent surveys of battered women programs have found that although program staff recognizes that substance use problems are common among their clients, these programs have limited substance use assessments and interventions in place and only a minority of staff has substance abuse training. Further, a huge challenge for these battered women shelters is the lack of financial resources to provide substance use services to their residents, especially with current decreases in their federal and local funding and increases in demand for their services. A computer-delivered intervention for battered sheltered women with substance use problems is a novel approach that can overcome existing obstacles to addressing substance use in battered women shelters, particularly time, training, and cost limitations.

The proposed study will develop and assess an innovative, easily implementable, low-cost, computer-delivered intervention, the SHE Program (Safe and Healthy Experiences) that will address known barriers in early identification and intervention for battered sheltered women with substance use issues. The computer-based SHE Program will be based on motivational interviewing a well-defined intervention strategy that has yielded particularly promising results in a range of clinical issues and a range of patient populations, including substance using women and women with IPV. Motivational interviewing with its non-confrontational and collaborative approach and its emphasis on increasing autonomy, self-efficacy, and skill sets is consistent with an empowerment model; a highly recommended intervention model for victimized women.

**The pilot study aims are to:**

1. Develop our proposed preliminary computer-based intervention, incorporating information gained in focus groups.
2. Perform a small open trial (N = 15) of SHE to assess feasibility of recruitment of target population and acceptability of intervention and study procedures.
3. Conduct a randomized pilot trial in a sample of 60 battered sheltered women who report substance use problems within the three months prior to entering the shelter, to demonstrate the feasibility of SHE and the acceptability of SHE via participant report of ease of use, helpfulness, and overall satisfaction.
4. Examine preliminary evidence that relative to control condition, the computer-based intervention (SHE) will result in improvements in our primary outcomes of substance use (heavy drinking or drug using) days over a 6-month post-shelter period and in our secondary outcomes of greater use of substance use services (both treatment and self-help utilization) and reduced IPV severity over a 6-month post-shelter period. Theorized mediators include readiness to change and self-efficacy for behaviors related to substance use.

**Background**

Violence against women is a significant public health problem. Intimate partner victimization (IPV) is defined as violence by an intimate partner that may involve physical altercations (e.g., such as hitting, slapping or kicking), emotional or physical threats, and/or forced sexual relations. IPV is a widespread and significant public health problem. Approximately 5.3 million adult women in the U.S. experience victimization annually, which results in nearly 2 million injuries and 1,300 deaths^[^[^13^](#_ENREF_13)^]^; one of the leading causes of injuries to women^[^[^14^](#_ENREF_14)^]^. The degree of morbidity associated with IPV is reflected in the fact that it negatively affects eight of the ten leading health indicators identified by the Department of Health & Human Services (DHHS^[^[^13^](#_ENREF_13)^]^). Estimates of the medical cost burden of IPV range from USD 2.3 billion to USD 7.0 billion annually^[^[^13^](#_ENREF_13)^,^[^15^](#_ENREF_15)^]^. Further, there is accumulating evidence to suggest a significant relationship between maternal IPV and maternal child abuse and maltreatment^[^[^16^](#_ENREF_16)^,^[^17^](#_ENREF_17)^]^.

In recognition of the significant social problems posed by IPV, battered women shelters, which provide physical safety, as well as emotional support, are prevalent throughout the country. There are approximately 2,000 community-based shelter programs throughout the US, providing emergency shelter to approximately 300,000 women and children each year^[^[^18^](#_ENREF_18)^]^. Considering the number of women who seek help from these facilities and that sheltered battered women have already instituted a change in their life, a prime time to intervene may be while these women are already seeking help from shelters.

Substance use problems are highly prevalent in battered women*:* Lifetime prevalence rates for substance abuse or dependence are twice as high for women with IPV than women in the general population^[^[^19^](#_ENREF_19)^,^[^20^](#_ENREF_20)^]^ and battered women are 5.6 times more likely to develop a substance use disorder compared to women not exposed to IPV^[^[^19^](#_ENREF_19)^]^. For women in U.S. domestic violence shelters, it has been estimated that 42% are substance users^[^[^21^](#_ENREF_21)^,^[^22^](#_ENREF_22)^]^. Moreover, given the multitude of competing concerns and high degree of stress faced by battered women in shelters and when they leave shelters, women with prior substance use problems appear to be at high risk for relapse. Common theories to explain the strong association between these two phenomena have included the self-medication hypothesis, that is, IPV victims use substances to cope with the effects of violence^[^[^23-26^](#_ENREF_23)^]^. Conversely, substance use can impair a woman’s judgment or compromise her ability to move to safety (see review^[^[^27^](#_ENREF_27)^]^). A third possibility is that substance use may increase conflict in relationships, leading to violent behavior towards the woman, especially if her partner is also using alcohol or drugs^[^[^25^](#_ENREF_25)^,^[^28^](#_ENREF_28)^]^. The pathways linking IPV and substance use are complex. In general research supports a strong bidirectional relationship between these two morbidities.^[^[^26^](#_ENREF_26)^,^[^29-32^](#_ENREF_29)^]^ A longitudinal study of a national probability sample of 3,006 women followed for 2 years identified a cyclic relationship where drug use increased the risk of IPV and IPV increased the risk of both drug and alcohol use^[^[^26^](#_ENREF_26)^]^.

Recent research has demonstrated that comorbid substance use increases risk of new episodes of violence for women with IPV: In their comprehensive model of variables associated with a battered woman’s risk for future IPV, Foa et al. suggested that negative consequences of IPV such as substance use can, in turn, impede a women’s ability to curtail future violence^[^[^33^](#_ENREF_33)^]^. Consistently, longitudinal studies have found that substance use is associated with new episodes of abuse in battered women^[^[^24^](#_ENREF_24)^,^[^34^](#_ENREF_34)^]^. Furthermore, drug use is related to increased severity of physical partner victimization^[^[^35^](#_ENREF_35)^]^. Given that cessation of violence is necessary for recovery from its traumatic effects, it seems imperative to address victimized women’s substance use difficulties. Such an intervention may improve battered women’s ability to effectively use resources to help break the cycle of violence and establish their and their children’s safety.

Why shelter stay is an opportune time to address substance use issues*:* There is consensus among experts that a critical time to provide substance use interventions for women with IPV is when they are seeking shelter services, because at these times women are open to changes in their lives and identities^[^[^36^](#_ENREF_36)^]^ and most likely to recover from violence and from substance use^[^[^36^](#_ENREF_36)^,^[^37^](#_ENREF_37)^]^. In general, the current literature supports interventions that target both IPV and substance use^[^[^38^](#_ENREF_38)^,^[^39^](#_ENREF_39)^]^. The Substance Abuse and Mental Health Services Administration (SAMHSA) in the United States has identified service system integration as a key domain to improving services for women with substance use and violence^[^[^40^](#_ENREF_40)^]^ in their lives and have highlighted the importance of the need to involve diverse organizations, such as domestic violence programs in these integration efforts.

Despite the need, few battered women shelters address substance use issues among their residents. Unfortunately, few battered women shelters address substance use issues, even though substance use is extremely prevalent in this population^[^[^41^](#_ENREF_41)^]^. A survey of battered women shelters, found that although the program staff recognize that substance abuse problems are common among their clients, these programs have limited substance use assessments and interventions in place, and only a minority of staff have training in substance use issues^[^[^22^](#_ENREF_22)^]^. Furthermore, a huge challenge for these battered women shelters is the lack of financial resources to provide these substance use services to their residents^[^[^42^](#_ENREF_42)^]^, especially with current decreases in their federal and local funding and increases in demand for their services^[^[^43^](#_ENREF_43)^]^.

Our proposed project, will develop and assess an innovative, high-reach, easily implementable, low-cost computer-delivered intervention (Safe and Healthy Experiences; The SHE Program) that will address known barriers in early identification and intervention with sheltered battered women with substance use problems. Given the number of battered women shelters in the country, our proposed intervention, SHE, has the potential to impact a relatively large community of women and children and to significantly reduce the suffering associated with IPV and substance use. A computer-delivered intervention, if proven effective, can be widely disseminated while maintaining treatment fidelity across sites and may hold promise for other IPV populations with substance use issues.

The overall goal of the SHE Program will be to facilitate self change as well as treatment and community resource engagement with respect to substance use issues. The content of SHE will be theory-driven, consistent with a motivational interviewing (MI) model of behavior, and consistent with the literature on effective interventions for our target population and targeted risk factors. MI^[^[^56^](#_ENREF_56)^,^[^57^](#_ENREF_57)^]^ is a well-defined intervention approach, has been used to reduce drug use among non-treatment seekers^[^[^54^](#_ENREF_54)^,^[^58-61^](#_ENREF_58)^]^, has wide dissemination, and demonstrated efficacy across a range of behavioral areas^[^[^62^](#_ENREF_62)^]^ including substance use^[^[^63^](#_ENREF_63)^]^ and across a range of populations including high-risk women^[^[^64^](#_ENREF_64)^]^. Consistent with the Transtheoretical Model (TTM^[^[^65^](#_ENREF_65)^]^), MI utilizes stages and processes of change, evolving readiness and self-efficacy to change. Ambivalence about change is considered normative within the motivational interviewing framework. The client’s readiness to make changes is not assumed. Instead, an important exercise in MI is the exploration of level of readiness to change. The intervention is appropriate for varying levels of readiness to change. MI facilitates internal motivation to change through alignment of behavior change with deeply held beliefs, values, and goals.

The MI model is of particular value for substance using women with IPV in reducing their risks because MI has a non-confrontational and collaborative approach, emphasizes increasing a participant’s awareness to successful steps towards their own well-being, identifies participant’s strengths, and builds upon participant’s successes. Such an approach is consistent with recommendations for brief interventions in substance users^[^[^66^](#_ENREF_66)^]^ and for women with IPV^[^[^67^](#_ENREF_67)^]^. In fact, recently, the World Health Organization^[^[^68^](#_ENREF_68)^]^ has recommended a MI-consistent approach for intervening with women who have experienced IPV. Further, a MI approach is in keeping with the empowerment model, a highly recommended intervention model for women with interpersonal violence^[^[^69^](#_ENREF_69)^]^. The MI and IPV Workgroup (2010)^[^[^67^](#_ENREF_67)^]^ have suggested that the empowerment model and MI models converge around important principles, such as increasing autonomy, self-efficacy, and skill sets.

**Summary**

Our proposed project will develop and assess an innovative, easily implementable, low-cost computer-delivered intervention, the SHE Program, that will address known barriers in early identification and intervention with sheltered battered women with substance use issues. Considering the number of women who seek help from battered women shelters and that sheltered battered women have already instituted a change in their life, battered women shelters is a prime setting for a substance use intervention. Further our proposed brief computer-delivered intervention is well suited to battered women shelters because it addresses the limited time, resources, and training of shelter staff in providing substance use interventions. Finally, a computer-delivered intervention, if proven effective, can be widely disseminated while maintaining treatment fidelity across battered women shelters and may hold promise for other battered women with substance use issues in other settings.

**Methods - General**

Subjects and Recruitment Site

Participants for the RCT and Open Trial will be recruited from the Women’s Center of Rhode Island (WCRI), Sojourner House, New Hope, and Battered Women’s Shelter of Summit and Medina Counties. Participants eligible for the screening portion of the study will be residents at the women battered shelter, the Women’s Center of Rhode Island (WCRI),Sojourner House, New Hope, or Battered Women’s Shelter of Summit and Medina Counties. Shelter staff will inform research staff as new residents are admitted to the shelter. The research assistant (RA) will attend house meetings and schedule times to be at the shelters when new residents are available and will provide a description of the initial study phase, the screening phase (Phase A). Research assistants will also give shelter staff business cards to refer clients to the study. Shelter residents will be recruited to participate in a computer-based survey to help battered women be healthy. Women who express interest will be given an information sheet (used to protect anonymity of those who did not qualify or chose not to participate in the full study). The information sheet will indicate that the purpose of screening is to find women who may be eligible for a study on risk factors for battered women and will describe what is involved with participating in the study. Women who verbally agree to participate will be given the opportunity to provide written consent to contact to set up a future appointment with the research staff. Women who refuse to complete screening for the study, or if after completing screening refuse to participate in the study, will be asked by the research assistant about possible reasons for refusal. Women who are interested will be screened for eligibility using the CIAS software delivered on an easy to use, ultraportable Tablet PC in a 10-15-minute screener (there will be no identifiers at screening) or a computer in a private setting. The use of Audio Computer-based Assisted Self-Interview (ACASI) software to conduct screening will maximize identification rates of women at risk for substance use. The screener will include a brief series of questions about general health, exercise, and diet. The screener will also include basic demographic questions as well as the following well-validated and reliable measures for this population: The NIDA-Modified Alcohol, Smoking, Substance Involvement Screening Test (NIDA-Modified ASSIST^[^[^78^](#_ENREF_78)^]^) will be used to assess for alcohol use, illegal drug use, and nonmedical prescription drug use in the past three months prior to shelter stay. Those who will be fully eligible for the study (and considered as at risk) will be categorized as an at-risk drinker and/or at moderate or high risk for substance use based on the NIDA-Modified ASSIST^[^[^78^](#_ENREF_78)^]^ criteria for these risk categories. Women who have a prescribed medical marijuana card will be excluded from the study. The screener will also include a well-validated, reliable and recommended IPV measure. At screening, IPV in the past three months prior to shelter stay will be assessed with the Woman Abuse Screening Tool (WAST^[^[^79^](#_ENREF_79)^]^). The WAST is an 8-item instrument that measures physical, sexual, and emotional abuse and is consistent with the definition of IPV as defined by The American College of Obstetricians and Gynecologists^[^[^79^](#_ENREF_79)^,^[^80^](#_ENREF_80)^]^. It has correctly classified 100% of nonabused women and 92% of abused women in a known-group analysis^[^[^79^](#_ENREF_79)^]^, has good internal reliability^[^[^81^](#_ENREF_81)^]^, and has adequate concurrent validity, even with ethnic minorities^[^[^81^](#_ENREF_81)^]^. Consistent with similar studies, IPV status will be positive if a woman obtains a score of 4 or more on the WAST.

All participants screened will receive a standard health information brochure with further information and resources on the health topics mentioned, including a list of local substance use treatment referrals and community resources. Those meeting full inclusion criteria will be asked to provide signed informed consent and complete the computer based baseline assessments. All participants will be advised to keep their copy of the consent form in a secure location, or to discard this form if it is unsafe for them to keep it.

*Inclusion criteria***:** Women who are 18 or older, residents of a battered women’s shelter (the Women’s Center of Rhode Island [WCRI],Sojourner House, New Hope, or Battered Women’s Shelter of Summit and Medina Counties), who are at risk substance users within the 3 months prior to entering the shelter, as determined by the screener, the NIDA-Modified ASSIST,^[^[^78^](#_ENREF_78)^]^and endorse IPV within the 3 months prior to entering the shelter, as determined by the screener, the WAST^[^[^79^](#_ENREF_79)^]^

*Exclusion criteria***:** includes: 1) inability to provide informed consent (e.g., due to florid psychosis or other clear cognitive impairment), 2) inability to understand English (understand the consent form when read aloud.

**Study Procedure**

The research program for this R34 Exploratory Research Award will be divided into three phases. **Phase I** (the development phase) will consist of manual development, focus groups, and the open trial. During this phase, we will develop our computer based intervention, SHE, which will be informed by focus groups. We will also conduct a nonrandomized trial with 15 women (open trial) to gain experience with the screening, assessment and intervention, which will inform subsequent refinement of protocol and procedures following the guidelines for Stage I treatment development^[^[^73^](#_ENREF_73)^]^. The proposed study will use a sophisticated intervention development tool, CIAS developed by Dr. Steven Ondersma, that has been successfully used with perinatal substance using women^[^[^11^](#_ENREF_11)^,^[^12^](#_ENREF_12)^]^. The Co-I (Dr. Tzilos) has been trained by Dr. Ondersma to use this software and successfully developed and tested an intervention to reduce alcohol use in an urban setting, for high-risk pregnant women^[^[^10^](#_ENREF_10)^]^. During **Phase II** (the pilot study phase), we will conduct a **two-group**, **randomized controlled study** with a sample of 60 women endorsing inclusion criteria (see Section C.2.3). Study participants will be randomly assigned to either the SHE or a time-and-attention-matched control condition to assess the feasibility of the research design, the acceptability of SHE, and to provide some preliminary information about the direction of intervention effects for substance using days over a 6-month post-shelter period (primary outcome) and total days of substance use services received and IPV severity over a 6-month post-shelter period (secondary outcomes). During **Phase III** **(Revision Phase),** the team (i.e., Drs. Zlotnick, Tzilos, D Johnson, and J Johnson) will review experiences and outcomes for both the open trial and the pilot study. Results from this proposal will be used as preliminary data for a fully-powered, larger clinical trial.

The SHE Program will consist of a 50 minute intervention on the Tablet PC. There will be a 15-minute “booster” session on the Tablet PC within 3 weeks after the 50 minute intervention session to reinforce the effects of the intervention. Both sessions will take place in a private shelter setting while the participant is a resident of the shelter. The SHE Program will follow the guidelines for brief interventions and contain the recommended elements for brief interventions: feedback of risk that is personalized (F), emphasis on personal responsibility (R), non-confrontational advice to change (A), a menu of change options (M), empathy as a counseling style (E), and enhancement of self-efficacy (S)—or FRAMES^[^[^70^](#_ENREF_70)^]^. Motivational strategies such as reflective listening and tailored summaries and feedback will be utilized to enhance participants’ motivation.

Some aspects of MI (and FRAMES model) are difficult to translate *literally* into a computer-based intervention (e.g., empathy). The software relies heavily on realistic interactions with a three-dimensional animated narrator to mimic a non-judgmental, empathic, and non-threatening demeanor of a person-delivered brief, motivational interventions. The narrator reads each item for the participant, and guides her throughout the process using reflections and self-deprecating humor. Throughout the development phase of the study, we will receive feedback on participants’ sense of autonomy, of being supported and reinforced in their decisions, and on the tone of the module. The software, including the three-dimensional animated narrator, has been found in previous research to be well-understood and well-liked by low-income women.^[^[^10^](#_ENREF_10)^,^[^12^](#_ENREF_12)^]^.

The computerized intervention will begin with a baseline **assessment** of substance use Participants will then receive immediate “profiles” **(personalized feedback)** based on their answers to assessments. A substance use profile will summarize their typical pattern of substance use, their substance use risk level, the known health and societal consequences associated with their level of substance use, and the negative consequences they identified as resulting from substance use. Next there will be an **education** component that will deliver facts about the woman’s substance use, and associated risks for the woman, including partner substance use; the **bidirectional relationship** between substance use and IPV; and risks of untreated substance use such as increased risk of IPV.

A limitation of some clinical trials of MI-based interventions for substance use and most clinical trials of behavioral interventions for IPV^]^ is the imbalance in intensity of sessions between the experimental intervention and control group, which makes it difficult to determine if intervention differences are attributable to differences in attention between the conditions or to the “active ingredients” of the intervention. Because the proposed non-MI control condition will be matched for duration, level of interactivity, and mode of delivery and has been shown to be a credible control condition^[^[^10^](#_ENREF_10)^]^, it will provide a reasonable test of the effect of the SHE Program. Furthermore, our control condition will have no overlapping content with the SHE Program. Finally, since both conditions will be computer-based of similar duration, the blinding of research staff to experimental condition will be facilitated.

There are no standard or universal procedures in place for screening and brief interventions for women with IPV and substance use issues. Like most battered women shelters^[^[^41^](#_ENREF_41)^]^, WCRI does not conduct any formal or systematic substance use-related assessments or interventions for their residents, besides appropriate referrals for substance use treatment, if requested, or if needed (i.e., resident discloses difficulties or is found to be using alcohol or drug during her shelter stay). All participants who consent for the RCT study (and open trial) will receive a list of appropriate substance use treatment referrals and community resources (e.g., NA, AA) and a brochure on alcohol and drug risks.

The intervention will be tailored on the current substance use status of each participant (see Figure 1). Computer software can easily deliver such a bifurcated treatment approach – such an intervention design is consistent with evidence that motivational approaches may work best with less motivated individuals^[^[^71^](#_ENREF_71)^]^. The intervention will contain language referring to either substance use in general (past or present behaviors and beliefs) or current substance use, and will not assume current substance use. For women who state that they have already quit (see “No” in Figure 1), the three-dimensional animated narrator will present a section that will focus on how they can remain abstinent now and after they leave the shelter (Relapse Prevention). An example in this section will include if the woman decides to return to her abuser, how avoiding using with her abuser could decrease her risk for abuse and for subsequent substance use. These women (i.e., those who have already quit) will have the option to create a personalized **Safety Plan**, and can select from a menu of potential personal change goals (i.e., maintain their change) and/or optionally, they can enter their own change goals in free text.

Women who endorse current substance use will be asked about their interest/readiness in quitting their substance use (“Ready to Quit?”) leading to a bifurcated treatment response such that those participants reporting a goal of immediate abstinence will move more quickly to a section consistent with primary goal-setting for substance use reduction. This arm will follow closely the guidelines established in previous intervention studies^[^[^72^](#_ENREF_72)^]^ which will include components of a quit contract and will assist the woman in identifying specific goals and a timeline for reaching these goals. These participants will have the option to create a personalized **Safety Plan** that will include the specific goals that the women have identified and/or optionally, can enter their own change goals in free text. Some example of goals will include attending AA or NA, attending substance use treatment, speaking to shelter staff about coordinating substance use treatment, planning for support groups after leaving the shelter such as IPV support groups. For the remaining participants who report not being ready to quit at this time, the narrator will present sections including **pros and cons, feedback, and optional goal-setting**. Specifically, in the pros and cons section, the narrator will normalize feelings of ambivalence about substance use, noting that most people can identify sides to their use that are both positive and negative. For example, the narrator will first ask for the good things about substance use and provide a checklist in which the participant endorses as many pros as they would like. The narrator then notes that for many people there are also some “not so good” aspects to substance use, and presents another checklist in which the participant endorses as many cons of substance use as they would like. Then the narrator reflects back to them the specific pros and cons that they selected and notes that feeling two ways about one’s substance use is quite normal. For example, the narrator would say, “You really seem to feel two ways about this. On the one hand, you like the way that smoking marijuana helps you relax when you have reminders of the abuse. But at the same time, it is only a temporary fix for your problems.” In the feedback section, the narrator will first present participants with normative feedback regarding their typical substance use using an easy-to-read graphical display and clear description of what the feedback means. This feedback includes key pieces of information: how much the participant reported using, how much they think most other women use, and how much of it other women actually use. Normative feedback is followed by feedback with the specific negative consequences that the participants reported having experienced as a result of their substance use. The narrator will then elicit brief elaborations of negative consequences using simple questions keyed to each type of consequences. For example, if a woman endorsed the item regarding health problems as a result of substance use, the narrator could ask, “What sorts of health problems have you had?” and provide a checklist of possible response. A follow up question could then ask the participant to choose ways in which their health could be improved if they did decide to make a change. There will be a section that will provide feedback on each participant’s self-reported readiness to change with regard to substance use. In the optional goal-setting section, the narrator will begin by briefly summarizing the state of each participant’s ambivalence regarding her substance use, and the factors pulling her in either direction. The narrator then asks if she would like to set a change goal. If she does not wish to do so, the narrator reflects her lack of readiness at this time and elicits information regarding what signs would tell the participant that she did need to change. If the participant does wish to set a change goal, the program will guide her through a brief change plan process. In this process, the participant will be asked when, why, and how she would like to make a change regarding her substance use and be given the option to create a personalized **Safety Plan** that incorporates the changes the woman has identified and/or any other changes she would like to add in free text. All SHE participants will be provided with an optional one-page print-out of the Safety Plan as a resource.

Booster session: Within three weeks of the intervention, women will complete a 15 minute computerized **booster session** in which they will review the relevant components of the intervention session for each woman (e.g., pros and cons, feedback, and goal-setting) and their own **personalized safety plan**. The booster session will bolster the effects of the intervention. Women will be given the option to revise their personalized **Safety Plan**, and can select from a menu of potential personal change goals; they can enter their own change goals in free text. All participants will be provided with an optional one-page print-out of the Safety Plan as a resource.

**Methods - Open-Pilot Trial**

In Month 7, we will begin recruitment for the nonrandomized trial with 15 women to gain experience with the screening, assessment and intervention, and to inform subsequent refinement of protocol and procedures. After the baseline assessments have been completed, the study participant will complete the SHE Program (an initial session plus booster session within three weeks prior to leaving the shelter) and complete 3- and 6-month follow-up assessments. After the completion of the SHE Program, participants will complete a computer based intervention specific End-of-Intervention Questionnaire, which will address participants’ perceptions of the helpfulness of each intervention component, including timing and length of the sessions and their level of comfort with research procedures. Participants will discuss their responses at an end-of-intervention exit interview with Dr. Zlotnick or a trained research assistant to aid in intervention revisions.

**Methods - Randomized Pilot Trial**

Women who are residents of a battered women’s shelter (the Women’s Center of Rhode Island [WCRI],Sojourner House, New Hope, or Battered Women’s Shelter of Summit and Medina Counties), who are at risk substance users within the 3 months prior to entering the shelter as determined by the screener, the NIDA-Modified ASSIST^[^[^78^](#_ENREF_78)^]^, and endorse IPV within the 3 months prior to entering the shelter as determined by the screener, the WAST^[^[^79^](#_ENREF_79)^]^ (Phase A) will be eligible for the RCT (Phase B). Phase B will use a two-group, randomized, controlled design with 60 shelter residents. After eligible participants consent to participating in the RCT and complete the computer-based baseline assessments the (computer) narrator will “flip a coin” and randomly assign participants to the SHE Program (an initial session plus booster session within three weeks after the initial session) or to the time and attention matched control condition (an initial session plus a booster session within three weeks after the initial session; both consisting of video viewing of popular shows and preference ratings). Next study participants will complete their assigned computer-based intervention. There will be 3- and 6-month post-shelter computer-based follow up assessments (see figure 2 below).

**Figure 2: Methods for Phase B**

For those who have completed Phase A (i.e., the screener) and who are eligible (i.e., endorse at risk substance use and IPV in the three months prior to shelter stay), research staff will inform them about the clinical trial, explain the informed consent process, and explain the study procedures. Refusal rates will be recorded and residents who refuse participation will be asked to provide their reasons for refusal. Women who provide informed consent will be given the computer again, and complete an approximately 20-30-minute baseline assessment using the ACASI software. The informed consent process, the computer-based baseline assessments, and computer-based sessions for both conditions will be conducted in a private space at the shelter. Follow-up assessments will be conducted in a private room or at a location that is a convenient location for the woman and one that will provide a confidential, safe, and comfortable environment for the participant. If a follow-up assessment is being conducted at a woman’s home, the research team may ask her if she will allow the research team to use her internet connection to run the computer program on the Tablet PC. If a participant does not have internet access or does not allow the researchers to access her internet connection, the research team will use our own portable air card for wireless internet connection. Participants will be reminded that if they do not wish to use their internet connection, they can still participate in the study. All participants in this study will receive care as usual from shelter staff; no screening, referral, or counseling will be withheld in any way at any time.

**Randomization**

After the screening has been completed (Phase A), for those who are eligible who have provided informed consent, and who have completed baseline assessments, the (computer) narrator “flips a coin” and randomly assigns participants into control vs. SHE Program conditions (Phase B).

**The SHE Program condition**

SHE participants will receive a 50-minute intervention after randomization and a 15-minute booster session with three weeks after the initial session on the Tablet PC. (The research team may modify the length of intervention sessions based on open trial feedback). The computer-based intervention and booster session will be conducted in a private space at the battered women’s shelter during participants’ shelter stay. SHE will take place during participants’ shelter stay because women are more likely to initiate changes when they are in a supportive environment.

**Control condition**

Control group participants will complete the same assessment measures. After randomization, participants in the control condition will receive a 50-minute session and a 15-minute booster session within three weeks after the initial session on the Tablet PC, which will comprise of the viewing of popular entertainers/shows videos. See above for description of the control condition. This condition will control for time spent on the computer-based intervention, maintain blinding of research assistants, and mimic the interactivity of the computer-based intervention condition. The control condition will be conducted in a private space at the shelter during participants’ shelter stay.

**Feasibility**

Our target enrollment of 60 women within an 18 month period appears to be feasible because there are high rates of substance use difficulties among battered sheltered women. Researchers estimate that about 42% of residents of battered women shelters are substance users^[^[^21^](#_ENREF_21)^]^. Furthermore, we found in our study of a treatment for battered sheltered women with posttraumatic stress disorder, where women with active substance dependence were ineligible and less likely to be referred to the study, 24% (*n* = 27) of participants assessed at baseline (*n* = 112) still met full criteria for a substance use disorder in the last 3 months. Moreover, there is a relatively high volume of women residents at our recruitment site, and our use of a computer-delivered screener should facilitate disclosure^[^[^47^](#_ENREF_47)^]^.

**Measures**

Participants who screen positive for substance use problems and IPV within the 3 months prior to shelter stay during screening will complete an approximately 20-30 minute baseline assessment session on the Tablet PC. Screening measures for at risk substance use and IPV are the same as the open trial and include the NIDA-Modified ASSIST^[^[^78^](#_ENREF_78)^]^ and the WAST^[^[^79^](#_ENREF_79)^]^, respectively. Most measures for this study will be completed by participants using the Audio Computer-based Assisted Self-Interview (ACASI) software and the Tablet PC. If there are any technical issues with the Tablet PC, paper copies of the same measures will be offered to participants to complete. Like the data completed on the tablet, the paper versions of the measures will be de-identified data. Measures will be administered to both groups of women at baseline (i.e., after screening and consent) and at 3- and 6 months after shelter discharge. The baseline battery is designed to minimize assessment with the control group in order to take into account growing concerns regarding the motivational properties of assessment, leading to substantial Type II error in other MI-based intervention studies^[^[^94^](#_ENREF_94)^,^[^95^](#_ENREF_95)^]^.

*Demographic measures*: All participants will complete demographic information, including age, race, ethnicity, educational level, marital status, occupation, employment (status, # hours per week), socioeconomic status, etc. See Table 1 for the time schedule for the study assessments.

*Acceptability: Satisfaction with CIAS Software Scale (SSS)* assesses participant satisfaction on items tapping on likeability, ease of use, level of interest, and respectfulness^[^[^12^](#_ENREF_12)^]^. Participants will also complete the well-studied 8-item *Client Satisfaction Questionnaire-Revised (CSQ-8-R)* to assess satisfaction with the intervention^[^[^96^](#_ENREF_96)^]^. These scales will be administered after each computer-based session (i.e., initial session and booster session) and administered to both conditions.

The following measures will be administered to both groups of women at baseline and at 3- and 6- months after leaving the shelter.

*Primary outcome measures:* *Alcohol and Substance use:* *Timeline Follow-Back (TLFB)-modified computer version*^[^[^97-99^](#_ENREF_97)^]^ has yielded comparable results to face-to-face interview administrations. The computer-based TLFB will assess drug use and heavy drinking (4+ standard drinks) days for the past week and the past 90 days from entering in the shelter. The TLFB will be done in person as well. For primary analysis, days using drugs and heavy drinking days will be combined to create a single variable that reflects the total number of days that women used drugs or had 4+ drinks. The TLFB has excellent reliability^[^[^100^](#_ENREF_100)^]^, and is sensitive to change as used in this study^[^[^101^](#_ENREF_101)^,^[^102^](#_ENREF_102)^]^. (We will not collect collateral (i.e., from a significant other) reports of subjects’ use to corroborate yes/no use of substances because this could pose a risk to confidentiality for the participant, who might be particularly vulnerable if she is experiencing IPV). To provide objective corroboration of self-reported drug use, *hair sample testing* (Psychemedics, Inc) at the 3- and 6-month will be obtained to give an approximately 90-day window of evidence for cocaine, marijuana, amphetamine, or opiate use.

We will report correlations between self-report and biological measures of substance use. *Breath alcohol tests* will be conducted at each follow-up assessment (i.e., at the 3 months and 6 months periods in order to insure that the client has consumed no alcohol prior to the assessment (breath alcohol content < .02). If the participant has been consuming alcohol, the assessment will be rescheduled. Urine drug screen results will also be reviewed to corroborate self-report data at the 3- and 6- month assessments. Urine drug screens will use theCLIAwaived, Inc. Instant Drug Test Cup-7 Panel Cup (ICTC), be unobserved, and test for benzodiazepines, cocaine, methamphetamine, methadone, opioids, oxycodone, and THC. .

*Secondary Outcomes: The Treatment Services Review (TSR)*^[^[^103^](#_ENREF_103)^]^ will be used to assess total days of substance use services (both treatment and self-help utilization) received (including outpatient, day patient, residential treatment, NA, AA) to capture the extent to which women are reaching out to access recovery-related resources. At baseline and at each follow-up point, the TSR will assess total days of substance use services received for a 90 day window.

IPV: The Composite Abuse Scale (CAS) is a widely used self-report of behaviors scale with 4 subscales that measure severe, combined abuse, emotional abuse, physical abuse, and harassment. The CAS has recently been published in the Centers for Disease Control and Prevention compendium of intimate partner violence measures^[^[^120^](file:///\\cdsfsp10\Womens%20Issues%20Research\IPV%20shelter\Protocol\Open%20Trial%20and%20RCT\Final-Shelter%20Study%20Open%20%20RCT%20Protocol.docx)^]^. It consists of 30 items presented in a six point format requiring respondents to answer “never”, “only once”, “several times”, “monthly”, “weekly” or “daily” in a twelve month period ^[^[^121^](file:///\\cdsfsp10\Womens%20Issues%20Research\IPV%20shelter\Protocol\Open%20Trial%20and%20RCT\Final-Shelter%20Study%20Open%20%20RCT%20Protocol.docx)^]^.

The Cyber Stalking Scale measure is a 6 –item measure and assesses the use of technologies in stalking and harassment.

Safety Behavior Checklist (SBC)*^[^*[*^122^*](file:///\\cdsfsp10\Womens%20Issues%20Research\IPV%20shelter\Protocol\Open%20Trial%20and%20RCT\Final-Shelter%20Study%20Open%20%20RCT%20Protocol.docx)*^,^*[*^123^*](file:///\\cdsfsp10\Womens%20Issues%20Research\IPV%20shelter\Protocol\Open%20Trial%20and%20RCT\Final-Shelter%20Study%20Open%20%20RCT%20Protocol.docx)*^]^* has 15 items that assess the use of strategies suggested to keep victim safe (e.g., hiding money and extra clothing).

*Theorized Mediators: The Confidence Ladder* will be used to assess the participants’ levels of confidence regarding their ability (self-efficacy) to change and abstain from substances. *Readiness Ruler* will be used to assess the degree to which participants’ are ready to cut down or quit alcohol or drugs.

Women who participated in the SHE intervention will complete a computer-based measure at the 3- and 6-month follow-up assessment to determine the extent to which participants achieved their goals on their personalized plan; an indication of the degree to which the SHE intervention had an impact on behavioral change. This measure will follow a similar format to the Effectiveness in Obtaining Resources Scale*,* which assesses battered women’s effectiveness in obtaining various resources.

At follow up, if a participant is unable to meet in-person to complete the assessments (e.g. participant has moved out of state), she will be given the option of participating in the assessment over the phone. Phone sessions will consist of the research assistant administering all the follow up assessments listed above via a phone session, with the exception of breathalyzer, urine, and hair sample testing.

**Table 1: Schedule of Assessments:**

| Measure | Intake | Post  intervention and booster | 3-months after shelter discharge | 6 months after shelter discharge |
| --- | --- | --- | --- | --- |
| Demographics | X |  |  |  |
| Timeline Followback | X |  | X | X |
| Hair Sampling Testing |  |  | X | X |
| Breath Alcohol Testing |  |  | X | X |
| Urine Drug Screening |  |  | X | X |
| The Composite Abuse Scale | X |  | X | X |
| Safety Behavior Checklist | X |  | X | X |
| Treatment Services Review | X |  | X | X |
| Client Satisfaction Questionnaire |  | X |  |  |
| Satisfaction with CIAS Software Scale |  | X |  |  |
| Readiness Ruler | X |  | X | X |
| Confidence Ruler | X |  | X | X |
| Cyber Stalking Scale | X |  | X | X |

**Retention**

The PI has had high retention rates (+ 85%) in treatment trials involving difficult-to-follow populations (e.g., substance using women with interpersonal violence and women with IPV. Furthermore, our prior research with battered residents leaving shelter, our follow up rates have been 82% retention at 6 months post-shelter and 80% at 9 months post shelter. The current study will employ several approaches that we have found helpful in achieving these low attrition rates, including staff’s strong relationships with participants, efforts to value and appreciate the women’s participation in the study, and frequent personal contact with the women for the duration of the study. Research staff calls women to remind them of their appointments and maintain a list of two other people who will always know where the participant resides. Transportation is provided as deemed necessary to complete assessments. Further, compensation for participants’ time for assessments helps facilitate retention. Finally, Rhode Island’s small size and population stability (residents rarely move out of state) also facilitate participant retention.

**Limitations**

The limitations of this study include: potential issues related to disclosure of sensitive information, the use of self-reports, assessment reactivity and effect on measured outcomes, the inclusion of only English-speaking participants, and generalizability of study results to other substance using populations.

**Potential risks**

There are four potential low- to moderate-risks to subjects associated with this research project:

*Breach of confidentiality***:** Assessment procedures could reveal sensitive information about participants’ substance use and history of IPV. Risk of breach of confidentiality is possible, though highly unlikely. Specifically, if a participant tells research staff that she is planning to harm herself or her children, the research staff will report this information to the appropriate agency, as required by law. Other than the need to report those incidents that are regulated by mandatory reporting laws, we feel that there is minimal risk to participants with regard to other breaches in their confidentiality.

*Coercion***:** Coercion occurs when potential participants feel compelled to participate in research for reasons such as perceived demand or the availability of large sums of reimbursement. This can be particularly true when there is little benefit to the individual for their participation (not an issue in this study). In the present study, the inclusion of a protected population and protection from coercion is of the utmost importance

*Discomfort***:** Participation in the study may lead to psychological distress due to the sensitive nature of questions regarding disclosure of IPV and substance use and the related negative social and psychological consequences.

*Study-related partner violence***:** Participants who return to or continue to have contact with their abuser may be at increased risk for abuse if he or she were to find out about the woman’s participation in the research project.

Social or legal consequences: Possible social or legal consequences due to revelations of IPV.

**Adequacy of protection against risks**

At each point of contact in the studies, participants will be reminded of the alternative of not participating in the study (or once enrolled, to discontinue participation), and will be informed that their care at the Women’s Center, Sojourner House, New Hope, or Battered Women’s Shelter of Summit and Medina Counties, and any other follow-up care will in no way be affected by their decision to participate or not to participate in the study. Moreover, we will provide referral information to all participants at each point of contact. Further procedures to minimize each of these risks are described below.

*Recruitment and informed consent:* Shelter staff will inform research staff as new residents are admitted to the shelter. The RA will attend house meetings and schedule times to be at the shelters when new residents are available and will provide a description of the initial study phase, the screening phase (phase A). Shelter residents will be recruited to participate in a computer-based survey to help battered women be healthy. Women who are interested in participating in the screener will receive an information sheet, which will be summarized (and read for those who prefer this) in a private setting. The information sheet (used to protect anonymity of those who did not qualify or chose not to participate in the full study) will indicate that the purpose of screening is to find women who may be eligible for a study on health risk factors and will describe what is involved with participating in the study. Those women who verbally agree to participate will complete the 10-minute screener (there will be no identifiers at screening) using the CIAS software delivered on an easy to use, ultraportable Tablet PC. Women meeting study screening criterion (i.e., endorse substance use in the three months prior to shelter stay) will be given the opportunity to provide informed consent for the clinical trial, in a private setting. Research staff will carefully explain all aspects of the study to a potential recruit, including the risks and benefits and obtain participants’ written informed consent. Recruits will be informed of the intervention commitment, amount and general types of assessments, and the follow-up procedures. The research assistant will orally describe the material written in the informed consent document and answer any questions the participant may have. Participants will be reminded that they are not required to participate in the study and that they will receive the standard care provided by the Women’s Center, Sojourner House, New Hope, or Battered Women’s Shelter of Summit and Medina Counties regardless of whether or not they choose to participate. Participants who give their consent will sign a copy of the document and will be given a signed copy of the informed consent document. Women who refuse to participate in the clinical trial will be provided referral information.

The use of computer technology for gathering self-report data further enhances overall protection. Data will be encrypted in transit between user and server. Importantly, no identifying information will be entered into the ACASI. Given the sensitive nature of this research, the computer software will simply generate a code number for each participant. Additionally, all forms with participant information will be marked with a code number and not with the participants’ name. The PI will keep the link between the participant code number and name under lock-and-key. Any information about a participant will never be released to outsiders without their explicit consent, except in the event of abuse of children/elderly/handicapped (report to the State of Rhode Island) or a medical emergency, when pertinent medical information will be given to the medical personnel caring for the individual.

**Protection against risk**

We will take the following steps to protect against risks associated with this research project:

*We will minimize the risk of breach of confidentiality***.**

A Certificate of Confidentiality will be obtained from the National Institutes of Health prior to the commencement of research. The purpose of this certificate is to protect the identity of research subjects participating in studies that collect sensitive information. Potential participants will be informed that a Certificate of Confidentiality has been obtained for this project and that this certificate will protect the investigators from being forced to release any research data in which participants can be identified, even under court order or subpoena, although this protection is not absolute. Potential participants will be informed of the situations in which they may not be protected under the Certificate of Confidentiality. No information about participants will be released without their permission or where required by law. Dr. Zlotnick has successfully used this approach in previous studies involving substance using women and women with intimate partner violence (IPV).

Possible distress due to sensitive items will be noted clearly in the informed consent information sheet (for screening) and in the written informed consent form (for participants). All participants will be told that they can skip any question or quit at any time if they become uncomfortable. Moreover, clinical backup will be provided during all assessments and during the intervention phase of the study by a licensed clinician to help facilitate any stabilization and referral process for participants who decompensate during assessment procedures. The need for additional services will also be monitored during all follow-up assessments.

To further address possible distress due to sensitive items, participants will be asked by the computer program if anything the computer has asked or done is making them feel upset right now. The computer program will notify the research team at completion of the screening, intervention session, or assessment, if any participant answers yes to this question. (Note that the computer program will not provide details regarding any answers, only that there is a need to follow-up with the participant verbally to evaluate the need for assistance). At a minimum, all participants indicating some distress will be given a list of referral options. For the duration of the study, women who report significant homicide or suicide risk will be immediately referred for evaluation for psychiatric admission at a local emergency department and if in the shelter, the appropriate staff will also be notified. All research personnel (i.e., interventionists, research assistants) will be trained in the protocol for homicidal or suicidal risk and research procedures for these situations. A licensed clinician will be available at all times by pager. Research staff will contact the licensed clinician if there are any safety concerns.

*Suicide or homicide risk*: All subjects will be closely monitored for significant suicidal and homicidal risks. During any phase of the study, if study interventionists, supervisors, or research staff judge that a subject is a significant suicide risk, the subject will be evaluated immediately by emergency room clinical staff at the closest hospital. If a subject reports active suicidal or homicidal ideation or plan during the SHE booster session, they will be evaluated individually by the study interventionist. If the suicidal or homicidal plan warrants immediate action, the interventionist will initiate voluntary or involuntary hospitalization. A licensed clinician will be available at all times by pager. Research staff will contact the licensed clinician if there are any safety concerns.

*Referrals for additional care:* All participants screened will receive a standard health information brochure with further information and resources on the health topics mentioned in the screening survey including a list of local substance use treatment referrals and community resources. Likewise, all participants who consent for the open trial or RCT study will receive a list of appropriate substance use treatment referrals and community resources. At any follow-up assessment, any participant who reports heavy drinking or the use of any illegal substances will be referred to appropriate treatment. Likewise, at follow-up assessments, any participant who reports current intimate partner violence (IPV) will be provided with information on IPV and a list of local IPV resources.

*We will minimize the risk of study-related partner violence***.** Participants who return to or continue to have contact with their abuser may be at increased risk for abuse if he or she were to find out about the woman’s participation in the research project. The measures suggested by Sullivan and Cain (2004) will be taken to maximize participant safety throughout the research. All phone calls will go through a confidential research line where the number is blocked. This line is answered, “Women and Infants Program,” and partner violence is never mentioned. Details of the research are not provided to anyone other than the actual participant. During each contact, participants will first be asked if it is a safe time to speak. Safe contact for follow-up assessments will be negotiated at each stage of the research. Both written and verbal contacts will be vague and never mention that the research involves partner violence. Follow-up interviews will be scheduled at a safe location. Safety plans will be negotiated up front (e.g., code words, cover story for reason for interview) if ever the abuser were to interrupt a phone call or assessment. Any handouts with important information (e.g., hotline numbers) will be available in wallet size copies without any reference to the study or name of organization. Participants’ safety contact information will be updated monthly to determine if the contact information they previously provided is still accurate and safe. Participants will be given contact information for the research team and asked to let us know if the contact information they provided is no longer safe. All research personnel (i.e., interventionist, research assistant) will be able to call the PI for advice on any case where there is a concern for the safety of the respondent and the PI will be available at all times by pager.

Although the proposed intervention seeks to help a woman to develop strategies for keeping herself safe, it is possible that a woman who participates in the study might be at increased risk of partner violence because of increased assertiveness. At each follow-up assessment, our assessment of community resource use will ask about participants’ visits to emergency rooms, health professionals, etc. *The Composite Abuse Scale (CAS)* will be administered to monitor abusive experiences in the context of a woman’s relationship since the last assessment. Women will be asked at follow up assessments (3- and 6-month) about any occurrences of hospitalizations for medical, psychiatric, or substance use issues or emergency room visits, and whether or not any of these events were related to IPV in order to better gather information regarding any potential serious adverse events. These events will be reported to the Data Safety Monitoring Board (DSMB).

For the duration of the study, if a woman discloses that she is in an abusive relationship, she will be provided with the battered women’s crisis line for emergencies, referrals to battered women’s shelters, and told how to obtain a restraining order. If the partner of the participant is also abusing her children, the research staff person involved will let the woman know that she as the mother is responsible for protecting her children and if her partner hurts them and she fails to call child welfare or the police, she could be charged with neglect and her children could be taken away. The research staff will encourage the woman to call child welfare herself as child welfare is more likely to view the woman favorably under these circumstances. The research personnel involved will provide the woman with the relevant phone number/s. The research personnel involved will let the woman know that she will be calling child welfare herself because it is the law. This same procedure will be followed for any other case of suspected child abuse.

**Potential benefits of the proposed research to the subjects and others**

The potential benefits to women participating in this study include close monitoring of participants’ alcohol and drug use as well as participants’ increased awareness of community resources for IPV and alcohol and drug-related issues. Half of the participants in this study will not receive any form of an “active” intervention, and thus are unlikely to receive any direct health benefit; however, women in both conditions will receive a list of local substance use treatment referrals and community resources. Half of the participants will receive a brief intervention designed to increase the likelihood of reductions in substance use and IPV, self-change and/or obtaining community resources for alcohol and drug use.

We believe that the risks to participants in this study are very low, particularly given the lack of connection between identifying information and data. We believe that the risks that are present are justified given the tremendous potential of this research to produce a replicable and low-cost motivational intervention that is appropriate for this group. Such an intervention, then, could potentially be presented to unprecedented numbers of persons in a way that is financially and logistically feasible, potentially leading to beneficial effects for large numbers of substance using women with IPV.

*Importance of the knowledge to be gained.* An exploratory approach towards the development of an effective brief computer-based intervention for sheltered battered women with substance use problems could increase the likelihood of these women accessing services for their substance use difficulties and reducing their substance use and IPV severity. Further, there is promising evidence that the application of brief interventions to substance using women offers substantial benefits to the woman. The paucity of research targeting substance use problems with battered sheltered women and the potential success of implementing a computer-based brief intervention in this population invites the proposed research investigation.

**Data Analysis:**

For this treatment development grant, assessment of feasibility and acceptability of the intervention and research procedures is the primary goal. Nonetheless, pilot data can be used to demonstrate whether the effects of treatment look promising across a set of outcome variables, to begin to examine distribution of outcome variables and ranges of correlations among variables across time to inform future analytic strategies, and to suggest, in concert with results from larger scale clinical trials in related fields, the range of effect sizes that would be reasonable to expect in a future trial. As a result, we will obtain the between treatment condition effect size estimates at each assessment (e.g., Cohen’s *d* or *h*) as well as the correlation between the same dependent variable at adjacent assessments. We anticipate we will have complete data on approximately 41 participants. Sample size guidelines for treatment development from Rounsaville et al.^[^[^114^](#_ENREF_114)^]^ recommend 15 to 30 participants per cell. Given that group means begin to stabilize by around 15, we believe a sample of ~20-21 in each condition should provide some information relevant to demonstrating potential promise for the intervention.

Primary analyses will be *intent-to-treat* (using data from all treatment enrollees). Secondary *completer analyses* (including only subjects who attend at least the computer-based session) will also be conducted. Analysis strategies used (hierarchical linear modeling [HLM]) can accommodate missing data, and can be used in a sample of 41 with a highly constrained covariance structure. Outcome variables may be transformed to improve normality if needed. If any outcome variable is too zero-inflated to be normalized through transformation, it will be analyzed with logistic regression or generalized estimating equation techniques in this small pilot study, and then with zero-inflated Poisson or negative binomial regression techniques in the subsequent fully-powered study. We will compare rates of missing data across conditions. Per recent clinical trials guidelines^[^[^115^](#_ENREF_115)^]^ analyses will adjust for baseline levels of dependent variables but will not test or adjust for any other baseline differences between conditions that result from randomization. Participants’ data will be directly exported from CIAS to a database immediately after each participant session. CIAS and ACASI data will be backed up daily and checked once per month for out of range values, missing data, and other quality issues.

Study Feasibility and Intervention Feasibility/Acceptability. One of the primary goals of a treatment development study is to demonstrate the feasibility of the proposed treatment and of the study and recruitment methods^[^[^4^](#_ENREF_4)^]^. As a result, we will assess the feasibility of the research procedures by examining study recruitment and refusal rates, participants’ willingness to be randomized, follow-up rates, and range of responses to study questionnaires. We will assess the feasibility and acceptability of SHE by examining rates of intervention attendance, and rates of intervention completion (both the intervention session and booster session for both conditions). For our rates of session completion, we expect at least 80% will complete both baseline and booster sessions based on our good attendance rates in our other IPV intervention studies^[^[^1^](#_ENREF_1)^,^[^87^](#_ENREF_3)^,^[^116^](#_ENREF_116)^]^. We will also examine reasons for termination for consistent patterns. We will examine the acceptability of both SHE and control conditions using data from *Satisfaction with CIAS Software Scale*^[^[^12^](#_ENREF_12)^]^ (i.e., an average score of 4 out of 5 on the CIAS satisfaction scale; at least highly satisfied and engaged with software) and CSQ-8-R^[^[^96^](#_ENREF_96)^]^ (i.e., above the average score of 27 on the CSQ-8-R)(see Section C.3.11). We will examine mean, mode, and median scores.

Other Primary Outcomes. (1) In addition to examining pre-post changes in the intervention condition, we will calculate the effect size and 95% confidence intervals for number of substance using days [including drug-using and heavy drinking days (4+ standard drinks)]. Exploratory tests for differences between conditions will use HLM, with using days in the 6 months prior to intake as a covariate. (2) Secondary Outcomes. We will examine pre-post changes in the intervention condition, and calculate between-groups effect sizes and 95% CIs for (1) total days of treatment received measured by the TSR, and for (2) reduction in IPV severity using the CAS. IPV severity will be operationalized as the sum of the number of abusive acts endorsed on the CAS; a scoring method found to provide a valid measure of abuse severity IPV^[^[^117^](#_ENREF_117)^]^. For exploratory tests for differences between conditions, we will use HLM with baseline scores as covariates.

Assessment of processes and individualized treatment response. This R34 treatment development study is underpowered to explore mediation effects (MacKinnon’s mediation power = 16%); a subsequent R01 will test the hypothesis that improvement in readiness to change and self-efficacy variables partially mediate the effects of SHE on our primary outcomes. Similarly, though a full test of moderation will be conducted in a subsequent R01, we will explore potential predictors of intervention response including IPV severity and baseline substance use severity, and will conduct preliminary examinations of large minority differences in this study.

**Phase III Refinement of SHE**

Based upon our experience during the Development and Trial Phase, evaluation of the program, and feedback from participants, the Investigator Team will revise the intervention. These revisions and the published qualitative and quantitative results of this study will represent the final products of this R34 and will be used in conjunction with information regarding the recruitment methods and retention of participants in the application for an R01 to evaluate this new intervention for our target population.

**Figure 3: Timeline for R34 Research Activities**

|  | **Year 1** | | | | | | | **Year 2** | | | | | | | **Year 3** | | | | | | |
| --- | --- | --- | --- | --- | --- | --- | --- | --- | --- | --- | --- | --- | --- | --- | --- | --- | --- | --- | --- | --- | --- |
|  | 1 |  | 7 | | 12 | |  | | | |  |  |  | 28 | | |  |  | | 36 | |
| **Develop intervention; conduct focus groups & qualitative analysis** |  | | |  | | | | | | | | | | | | | | | | | |
| **Conduct open trial, refine intervention** |  | | |  | | | | |  | | | | | | | | | | | | |
| **Recruit, randomize, & intervene** |  | | | | |  | | | | | | | | | |  | | | | | |
| **Collect post-release & follow-up assessments** |  | | | | | | | | |  | | | | | | | | | | |  |
| **Analyze data, prepare manuscripts, make final manual and scale revisions, write R01** |  | | | | | | | | | | | | | | | | | |  | | |

**Economic Considerations:**

Participants will receive gift cards and money (total equivalent up to $120) for their participation. Participants will receive a $5 gift card for completing screening, $30 for completing the baseline session and assessment before leaving the shelter, and $30 for 3 and 6 month follow-up assessments after leaving the shelter. Participants will also receive $5 for completing the booster session. Participants will receive an additional $10 for completing the hair sample testing. Cab transportation through a voucher system arranged with a local cab company will be provided for participants who may not own or have access to a car and need to have transportation in order to attend study procedures. Child-care expenses will be reimbursed to the study participants at a rate of $10 per hour for up to a 2 hour time period when applicable.

**Safety Monitoring Plan:**

An external Data and Safety Monitoring Board (DSMB) will be assembled to evaluate the data and safety to women enrolled in the study. The DSMB will consist of 4 senior doctoral-level/MD board members who have experience in clinical trials and/or IPV intervention research and/or research with substance using women as the ethical issues involved with a randomized controlled study, as indicated by peer-reviewed journal articles in these areas. We do not anticipate any difficulty in recruiting these qualified, independent board members as there is a pool of such researchers at several universities in Rhode Island and Massachusetts who are unaffiliated with Brown, who have the relevant experience.

The external DSMB will convene twice in Year 1, and then once during Year 2 for a meeting. Initially, the Board will convene with the PI to review the study protocol and review the guidelines for data and safety monitoring. This will include establishing standard procedures for daily (whenever there has been contact with a participant) and weekly monitoring by the local internal reviewers (PI and study personnel). At this meeting and at each subsequent meeting, the DSMB will evaluate recruitment, the progress of the trial, subject retention, data quality and confidentiality. In addition, they will review participants' clinical status, rates of adverse events and whether or not there have been any changes in risk to participating subjects. This review will ensure that subject risk does not outweigh study benefits. In the DSMB’s review of adverse events, if non-serious adverse events are occurring at a significantly higher rate in one condition than the other, then the DSMB will make appropriate recommendations for changes in the protocol, if needed. If Serious Adverse Events (SAEs) occur at a significantly higher rate in one condition than the other, then the DSMB might consider terminating the trial, if changes to the protocol are unlikely to address the high occurrence of the SAEs. We do not anticipate that this will occur, because we have taken several steps to avoid or protect against the occurrence of SAEs as outlined in the Section on Protection of Risk. A report generated from each of these meetings will be retained at the study site and will be forwarded to the hospital’s IRBs and to NICHD.

The DSMB will be available to convene outside of the appointed meeting schedule, if necessary, due to concerns regarding a particular subject, or due to any troublesome developments in subjects' experiences during the study. The DSMB will make appropriate recommendations for changes in the study protocol, if needed. The safety of participants will be monitored during each contact with study participants. Both anticipated and unanticipated adverse events and problems will be formally monitored and recorded. Unanticipated serious adverse events or problems will be reported to the hospital IRB and to NIDA within 48 hours.

In this study, we will use the FDA definition of serious adverse events (SAEs). RAs will report SAEs to the PI immediately. Data and safety of patients will be monitored by the PI. At a weekly meeting, the PI will review participants’ safety and will present participants’ clinical status and adverse experiences. Entrance criteria of all participants will be reviewed at these meetings. The PI will ensure that information on participants’ adverse effects are systematically collected and evaluated.

Dr. Zlotnick (or other key personnel if necessary) will immediately report any adverse events that are observed to the Women and Infants Internal Review Boards (IRB) and NIDA. The initial SAE report will be followed by submission of a completed SAE report to both institutions. Outcomes of SAEs will be periodically reported to NIH (NIDA). A summary of the SAEs that occurred during the previous year will be included in the annual progress report.

**Bibliography:**

1. Johnson, D. M. (2012). *Treatment of PTSD in residents of battered women’s shelters, in Beck, JG (Chair), Understanding Intimate Partner Violence: The Interplay of Research, Outreach, and Advocacy.* Paper presented at the 46rd Annual Convention of the Association for Behavioral and Cognitive Therapies, National Harbor, MD.

2. Zlotnick, C., Johnson, J., & Najavits, L. (2009). Randomized controlled pilot study of incarcerated women with substance use disorder and PTSD. *Behavior Therapy, 40*(4), 325-326.

3. Zlotnick, C., Capezza, N. M., & Parker, D. (2011). An interpersonally based intervention for low-income pregnant women with intimate partner violence: a pilot study. *Archives of Womens Mental Health, 14*(1), 55-65. doi: 10.1007/s00737-010-0195-x

4. Kraemer, H., Mintz, J., Noda, A., Tinklenberg, J., & Yesavage, J. (2006). Caution regarding the use of pilot studies to guide power calculations for study proposals. *Archives of General Psychiatry, 63*(5), 484-489.

5. Yeagley, E. K., Kulbok, P. A., O'Laughlen, M. C., Ingersoll, K. S., Rovnyak, V. G., & Rana, S. (2012). The feasibility and acceptability of a motivational interviewing intervention for HIV-infected youth in an urban outpatient clinic: a pilot study. *The Journal of Association of Nurses in AIDS care: JANAC, 23*(4), 366-370. doi: 10.1016/j.jana.2011.06.004

6. Dombrowski, S. U., Sniehotta, F. F., Johnston, M., Broom, I., Utkarsh, K., Brown, J., . . . Araújo-Soares, V. (2012). Optimizing acceptability and feasibility of an evidence-based behavioral intervention for obese adults with obesity-related co-morbidities or additional risk factors for co-morbidities: An open-pilot intervention study in secondary care. *Patient Education and Counseling, 87*(1), 108-119. doi: 10.1016/j.pec.2011.08.003

7. Berrouiguet, S., Gravey, M., Le Galudec, M., Alavi, Z., & Walter, M. (2014). Post-acute crisis text messaging outreach for suicide prevention: a pilot study. *Psychiatry Research, 217*(3), 154-157. doi: 10.1016/j.psychres.2014.02.034

8. Vanbuskirk, K. A., & Wetherell, J. L. (2013 Aug 11. [Epub ahead of print]). Motivational interviewing with primary care populations: a systematic review and meta-analysis. *Journal of Behavioral Medicine*.

9. Mello, M. J., Nirenberg, T. D., Longabaugh, R., Woolard, R., Minugh, A., Backer, B., . . . Stein, L. (2005). Emergency department brief motivational inteventions for alcohol with motor vehicle crash patients. *Annals of Emergency Medicine, 45*(6), 620-625. doi: 10.1016/j.annemergmed.2005.01.026

10. Tzilos, G. K., Solol, R., & Ondersma, S. (2011). A randomized phase I trial of a brief computer-delivered intervention for alcohol use during pregnancy. *Journal of Women’s Health, 20*, 1517-1424. doi: 10.1089/jwh.2011.2732

11. Ondersma, S., Svikis, D., & Schuster, C. (2007). Computer-based brief intervention: a randomized trial with postpartum women. *American Journal of Preventative Medicine, 32*(3), 231-238. doi: 10.1016/j.amepre.2006.11.003

12. Ondersma, S. J., Chase, S., Svikis, D., Lee, J., Haug, N., Stitzer, M., & Schuster, C. (2005). Computer-based brief motivational intervention for perinatal drug use. *Journal of Substance Abuse Treatment, 28*, 305-312. doi: 10.1016/j.jsat.2005.02.004

13. National Center for Injury Prevention and Control. (2003). *Costs of intimate partner violence against women in the United States*. Atlanta GA.

14. Tjaden, P., & Thoennes, N. (2000). *Full report of the prevalence, incidence, and consequences of violence against women: Findings from the National Violence Against Women Survey*. Washington, D.C.

15. Brown, D. S., Finkelstein, E., & Mercy, J. (2008). Methods for estimating medical expenditures attributable to intimate partner violence. *Journal of Interpersonal Violence, 23*(12), 1747-1766. doi: 10.11777/0886260508314338

16. Kelleher, K., Hazen, A., Coben, J., Wang, Y., McGeehan, J., Kohl, P., & Gardner, W. (2008). Self-reported disciplinary practices among women in the child welfare system: association with domestic violence victimization. *Child Abuse and Neglect, 32*(8), 811-818. doi: S0145-2134(08)00118-X [pii] 10.1016/j.chiabu.2007.12.004

17. Taylor, C. A., Guterman, N. B., Lee, S. J., & Rathouz, P. J. (2009). Intimate partner violence, maternal stress, nativity, and risk for maternal maltreatment of young children. *American Journal of Public Health, 99*(1), 175-183. doi: AJPH.2007.126722 [pii] 10.2105/AJPH.2007.126722

18. National Coalition of Domestic Abuse. (2006). Retrieved January 24, 2014, from <http://www.ncadv.org/>

19. Golding, J. (1999). Intimate partner violence as a risk factor for mental disorders: a meta-analysis. *Journal of Family Violence, 14*(2), 99-132.

20. Kessler, R., Sonnega, A., Bromet, E., Hughes, M., & Nelson, C. (1995). Posttraumatic stress disorder in the national comorbidity survey. *Archives of General Psychiatry, 52*, 1048-1060.

21. Bennett, L., & Lawson, M. (1994). Barriers to cooperartion between domestic violence and substance abuse programs. *Families in Society, 75*(5), 277-286.

22. Martin, L., Moracco, K. E., Chang, J. C., Council, C. J., Dulli, L. S., & (2008). Substance abuse issues among women in domestic violence programs: findings from North Carolina. *Violence Against Women, 14*(9), 985-997. doi: 10.1177/1077801208322103

23. El-Bassel, N., Gilbert, L., Wu, E., Go, H., & Hill, J. (2005). The temporal relationship between drug abuse and intimate partner violence: a longitudinal study among women on methadone. *American Journal of Public Health, 95*, 465-470.

24. Testa, M., Livingston, J., & Leonard, K. (2003). Women's substance use and experiences of intimate partner violence: a longitudinal investigation among a community sample. *Addictive Behaviors, 28*(9), 1649-1664.

25. Gilbert, L., El-Bassel, N., Rajah, V., Foleno, A., & Frye, V. (2001). Linking drug related activities with experiences of partner violence: A focus group study of women in methadone treatment. *Violence and Victims, 16*, 517-536.

26. Kilpatrick, D., Acierno, R., Resnick, H., Saunders, B., & Best, C. (1997). A 2-year longitudinal analysis of the relationship between violent assault and substance use in women. *Journal of Consulting and Clinical Psychology, 65*, 834-847.

27. Logan, T., Cole, J., & Leukefeld, C. (2002). Women, sex, and HIV: social and contextual factors, meta-analysis of published interventions, and implications for practice and research. *Psychological Bulletin, 128*(6), 851-885.

28. Goldstein, G. (1985). Institutional barriers to alcohol and drug abuse prevention. *The International Journal of the Addictions, 20*(1), 217-231.

29. Gutierres, S., & Puymbroeck, C. (2006). Childhood and adult violence in the lives of women who misuse substances. *Aggression and Violent Behavior, 11*(5), 497-513.

30. Nowotny, K., & Graves, J. (2013). Substance use and intimate partner violence victimization among white, african american, and latina women. *Journal of Interpersonal Violence, 28*(17), 3301-3318. doi: 10.1177/0886260513496903

31. Center for Disease Control and Prevention. (2010). *Intimate Partner Violence: Consequences*. Washington, D.C.

32. Center for Disease Control and Prevention. (2010). *Intimate Partner Violence: Risk and protective factors*. Washington, D.C.

33. Foa, E. B., Cascardi, M., Zoellner, L. A., & Feeny, N. C. (2000). Psychological and enviromental factors associated with partner violence. *Trauma, Violence and Abuse, 1*(1), 67-91. doi: 10.1177/1524838000001001005

34. Hedtke, K. A., Ruggiero, K. J., Fitzgerald, M. M., Zinzow, H. M., Saunders, B. E., Resnick, H. S., & Kilpatrick, D. G. (2008). A longitudinal investigation of interpersonal violence in relation to mental health and substance use. *Journal of Consulting and Clinical Psychology, 76*(4), 633-647. doi: 10.1037/0022-006X.76.4.633

35. Kubiak, S. P., Arfken, C. L., Boyd, C., & Cortina, L. M. (2006). More severe violence exposure associated with poly-pharmaceutical use. *American Journal on Additions, 15*(6), 457-461. doi: 10.1080/10550490600998583

36. Poole, N., & Greaves, L. (2008). Substance use by women using domestic violence shelters. *Substance Use and Misuse, 43*, 1129-1150. doi: 10.1080/10826080801914360

37. Zubretsky, T. M. (2002). Promising directions for helping chemically-involved battered women get safe and sober. In A. R. Roberts (Ed.), *Handbook of domestic violence intervention strategies*. New York: Oxford University Press

38. Afifi, T. O., Henriksen, C. A., Asmundson, G. J. G., & Sareen, J. (2012). Victimization and perpetration of intimate partner violence and substance use disorders in a nationally representative sample. *The Journal of Nervous and Mental Disease, 200*(8), 684-691. doi: 10.1097/NMD.0b013e3182613f64

39. Schneider, R., Burnette, M., Ilgen, M., & Timko, C. (2009). Prevalence and correlates of intimate partner violence victimization among men and women entering substance use disorder treatment. *Violence and Victims, 24*(6), 744-756.

40. Moses, D. J., Huntington, N., & D'Ambrosio, B. (2004). *Developing integrated services for women with co-occurring disorders and trauma histories*. Rockville, MD: Substance abuse and Mental Health Services Administration (SAMHSA).

41. Fowler, D. (2006). The extent of substance use problems among women partner abuse survivors residing in a domestic violence shelter. *Family and Community Health, 30*(1-Supplement), S106-S108.

42. Collins, J. J., Kroutil, L. A., Roland, J., & Moore-Gurrera, M. (1997). Issues in the linkage of alcohol and domestic violence services. In M. Galanter (Ed.), *Recent developments in alcoholism* (Vol. 13, pp. 387-405). New York: Plenum

43. Covert, B. (2013). Domestic violence shelters warn that sequestration has put women’s lives on the line. Retrieved January 21, 2014, from <http://thinkprogress.org/economy/2013/06/03/2093221/domestic-violence-shelters-fear-the-ultimate-cost-from-sequestration/>

44. Gilbert, L., El-Bassel, N., Manuel, J., Wu, E., Go, H., Golder, S., . . . Sanders, G. (2006). An integrated relapse prevention and relationship safety intervention for women on methadone: Testing short-term effects on intimate partner violence and substance use. *Violence and Victims, 21*(5), 657-672.

45. Smith, P., Homish, G., Leonard, K., & Cornelius, J. (2011). Intimate partner violence and specific substance use disorders: findings from the National Epidemiologic Survey on Alcohol and Related Conditions. *Psychology of Addictive Behaviors*. doi: 10.1037/a0024855

46. Coker, A., Davis, K., Arias, I., Desai, S., Sanderson, M., Brandt, H., & Smith, P. (2002). Physical and mental health effects of intimate partner violence for men and women. *American Journal of Preventative Medicine, 23*(4), 260-268.

47. Renker, P. R. (2008). Breaking the barriers: the promise of computer-assisted screening for intimate partner violence. *Journal of Midwifery & Women's Health, 53*(6), 496-503. doi: 10.1016/j.jmwh.2008.07.017

48. Cohen, R., & Stussman, B. (2005). Health information technology use among men and women aged 18–64: Early release of estimates from the National Health Interview Survey, January–June 2009. *Health E-Stats*.

49. Neighbors, C., Larimer, M., & Lewis, M. (2004). Targeting misperceptions of descriptive drinking norms: efficacy of a computer-delivered personalized normative feedback intervention. *Journal of Consulting and Clinical Psychology, 72*, 434-447.

50. Hester, R., Squires, D., & Delaney, H. (2005). The Drinker’s Check-up: 12-month outcomes of a controlled clinical trial of a stand-alone software program for problem drinkers. *Journal of Substance Abuse Treatment, 28*(2), 159-169.

51. Gilbert, P., Ciccarone, D., Gansky, S., Bangsberg, D., Clanon, K., McPhee, S., . . . Gerbert, B. (2008). Interactive “Video Doctor” Counseling Reduces Drug and Sexual Risk Behaviors among HIV-Positive Patients in Diverse Outpatient Settings *PLoS ONE, 3*(4). doi: 10.1371/journal.pone.0001988

52. Free, C., Phillips, G., Galli, L., Watson, L., Felix, L., Edwards, P., & Haines, A. (2013). The effectiveness of mobile-health technology-based health behaviour change or disease management interventions for health care consumers: a systematic review. *PLoS Medicine, 10*(1), 1-45. doi: doi:10.1371/journal.pmed.1001362

53. Glass, N., Eden, K., Bloom, T., & Perrin, N. (2010). Computerized aid improves safety decision process for survivors of intimate partner violence. *Journal of Interpersonal Violence, 25*(11), 1947-1964. doi: 10.1177/0886260509354508

54. Madras, B. K., Compton, W. M., Avula, D., Stegbouer, T., Stein, J. B., & Clark, H. W. (2009). Screening, brief interventions, referral to treatment (SBIRT) for illicit drug and alcohol use at multiple healthcare sites: comparison at intake and 6 months later. *Drug Alcohol Depend., 99*(1-3), 280-295. doi: 10.1016/j.drugalcdep.2008.08.003.

55. Woodruff, S. I., Eisenberg, K., McCabe, C. T., Clapp, J. D., & Hohman, M. (2013). Evaluation of California's alcohol and drug screening and brief intervention project for emergency department patients. *Western Journal of Emergency Medicine, 14*(3), 263-270. doi: 10.5811/westjem.2012.9.11551

56. Miller, W., & Rollnick, S. (1991). Motivational interviewing: Preparing people to change addictive behavior. *Motivational interviewing: Preparing people to change addictive behavior*.

57. Miller, W., & Rollnick, S. (2002). Motivational interviewing: Preparing people for change. *Motivational interviewing: Preparing people for change*.

58. Bernstein, J., Bernstein, E., Tassiopoulos, K., Heeren, T., Levenson, S., & Hingson, R. (2005). Brief motivational intervention at a clinic visit reduces cocaine and heroin use. *Drug and Alcohol Dependence, 77*(1), 49-59.

59. Bernstein, E., Edwards, E., Dorfman, D., Heeren, T., Bliss, C., & Bernstein, J. (2009). Screening and brief intervention to reduce marijuana use among youth and young adults in a pediatric emergency department. *Academic Emergency Medicine: Official Journal of the Society of Academic Emergency Medicine, 16*(11), 1174-1185. doi: 10.1111/j.1553-2712.2009.00490.x

60. Bernstein, E., Bernstein, J., & Levenson, S. (1997). Project ASSERT: An ED-Based Intervention to Increase Access to Primary Care, Preventive Services, and the Substance Abuse Treatment System. *Annals of Emergency Medicine, 30*(2), 181-189. doi: <http://dx.doi.org/10.1016/S0196-0644(97)70140-9>

61. D'Onofrio, G., & Degutis, L. C. (2010). Integrating project ASSERT: A screening, intervention, and referral to treatment program for unhealthy alcohol and drug use into an urban emergency department. *Academic Emergency Medicine: Official Journal of the Society of Academic Emergency Medicine, 17*(8), 903-911. doi: 10.1111/j.1553-2712.2010.00824.x

62. Burke, B., Arkowitz, H., & Menchola, M. (2003). The efficacy of motivational interviewing: A meta-analysis of controlled clinical trials. *Journal of Consulting and Clinical Psychiatry, 71*(5), 843-861.

63. Dunn, C., Deroo, L., & Rivara, F. (2001). The use of brief interventions adapted from motivational interviewing across behavioral domains: A systematic review. *Addiction, 96*, 1725-1742.

64. Weir, B., O'Brien, K., Bard, R., Casciato, C., Maher, J., Dent, C., . . . Stark, M. (2009). Reducing HIV and partner violence risk among women with criminal justice system involvement: a randomized controlled trial of two motivational interviewing-based interventions. *AIDS Behavior*, 509-522. doi: 10.1007/s10461-008.9422-0

65. Prochaska, J., & DiClemente, C. C. (1984). *The transtheoretical approach: Crossing traditional boundaries of therapy*. Homewood, IL: Dow Jones-Irwin.

66. DiClemente, C. (1986). Self efficacy and the addictive behaviors. *Journal Social and Clinical Psychology, 4*(3), 302-315.

67. Motivational Interviewing and Intimate Partner Violence Workgroup. (2010). Guiding as practice: motivational interviewing and trauma-informed work with survivors of intimate partner violence. *Partner Abuse, 1*(1), 92-104.

68. Feder, G., Wathen, C. N., & McMillan, H. L. (2013). An evidence-based response to intimate partner violence WHO guidelines. *JAMA, 310*(5), 479-480.

69. Dutton, D., & Hemphill, K. (1992). Pattens of socially desirable responding among perpetrators and victims of wife assault. *Violence Victims, 7*(1), 29-39.

70. Bein, T. H., Miller, W. R., & Tonigan, J. S. (1993). Brief interventions for alcohol problems: a review. *Addiction, 88*(3), 315-336. doi: 10.1111/j.1360-0443.1993.tb00820.x

71. Rohsenow, D., Monti, P., Martin, R., Colby, S., Myers, M., Gulliver, S., . . . Abrams, D. (2004). Motivational enhancement and coping skills training for cocaine abusers: effects on substance abuse outcomes. *Addiction, 99*, 862-874.

72. Ondersma, S. J., Svikis, D. S., Thacker, L. R., Beatty, J. R., & Lockhart, N. (2014). Computer-delivered screening and brief intervention (e-SBI) for postpartum drug use: a randomized trial. *Journal of Substance Abuse Treatment, 46*(1), 52-59. doi: 10.1016/j.sat.2013.07.013.

73. Onken, L. S., Blaine, J., & Battjes, R. (1997). Behavioral therapy research: a conceptualization of a process. In S. W. Henggeler, Santos, A.B., (Ed.), *Innovative approaches for difficult-to-treat populations* (1 ed., Vol. xxxiii, pp. 513). Washington, D.C.: American Psychiatric Association

74. Ondersma , S., Svikis, D., Lam, P., Connors-Burge, V., Ledgerwood, D., & Hopper, J. (2012). A randomized trial of computer-delivered brief intervention and low-intensity contingency management for smoking during pregnancy. *Nicotine and Tobacco Research: official journal of the society of research on nicotine and tobacco, 14*(3), 351-360. doi: 10.1093/ntr/ntr221

75. Kuo, C., Schonbrun, Y., Zlotnick, C., Bates, N., Todorova, R., Kao, J., & Johnson, J. (2013). A qualitative study of treatment needs among pregnant and postpartum women with substance use and depression. *Substance Use and Misuse*.

76. Kuo, C., Johnson, J. G., Rosen, R., Wechsberg, W., Gobin, R., Reddy, M. K., . . . Zlotnick, C. (2013). Women and health, emotional dysregulation and risky sex among incarcerated women with a history of interpersonal violence. *Women & Health*. doi: 10.1080/03630242.2013.850143

77. Draucker, C. B., Johnson, D. M., Johnson, N. L., Kadeba, M. T., Mazurczyk, J., & Zlotnick, C. (In press). Rapid HIV testing and counseling for residents in domestic violence shelters. *Women & Health*.

78. National Institute on Drug Abuse. (2009). Resource guide: Screening for drug use in general medical settings.

79. Brown, J., Lent, B., Schmidt, G., & Sas, G. (2000). Application of the Woman Abuse Screening Tool (WAST) and the WAST-Short in the family practice setting. *Journal of Family Practice, 49*(10), 896-903.

80. The American College of Obstetricians and Gynecologists. (2011). Domestic violence: frequently asked questions. *2012*. <http://www.acog.org/~/media/For%20Patients/faq083.pdf?dmc=1&ts=20120710T1451065788>

81. Fogarty, C., & Brown, J. (2002). Screening for abuse in Spanish-speaking women. *Journal of American Board of Family Practice, 15*, 101-111.

82. Satre, D., & Sterling, S. (2013). PS1-19: Impact of motivational interviewing to reduce alcohol use among depression patients. *Clinical Medicine and Research, 11*(3). doi: 10.3121/cmr.2013.1176.ps1-19

83. Bagoien, G., Bjorngaard, J. H., Ostensen, C., Reitan, S. K., Romundstad, P., & Morken, G. (2013). The effects of motivational interviewing on patients with comorbid substance use admitted to a psychiatric emergency unit - a randomised controlled trial with two year follow up. *BMC Psychiatry, 13*(93). doi: 10.1186/1471-244X-13-93.

84. Wilson, G. B. (2012). Brief intervention to reduce risky drinking in pregnancy: study protocol for a randomized controlled trial. *Current Controlled Trials in Cardiovascular Medicine, 13*(1). doi: 10.1186/1745-6215-13-174

85. Kiely, M., El-Mohandes, A., El-Khorazaty, M., & Gantz, M. (2010). An integrated intervention to reduce intimate partner violence in pregnancy. *Obstetrics & Gynecology, 115*(2, Pt. 1), 273-283.

86. McFarlane, J., Groff, J., O'Brien, J., & Watson, K. (2006). Secondary prevention of intimate partner violence a randomized controlled trial. *Nursing Research, 55*(1), 52-61.

87. Zlotnick, C., Capezza, N., & Parker, D. (2011). An interpersonally based intervention for low-income pregnant women with intimate partner violence: a pilot study. *Archives of Women's Mental Health, 14*(1), 55-65. doi: 10.1007/s00737-010-0195-x

88. Diefenbach, M. A., Mohamed, N. E., Butz, B. P., Bar-Chama, N., Stock, R., Cesaretti, J., . . . Hall, S. J. (2012). Acceptability and preliminary feasibility of an internet/CD-ROM-based education and decision program for early-stage prostate cancer patients: Randomized pilot study. *Journal of Medical Internet Research, 14*(1), 262-275. doi: 10.2196/jmir.1891

89. Berry, K., Purandare, N., Drake, R., Elmsley, R., Jones, L., & Barrowclough, C. (2014). A mixed-methods evaluation of a pilot psychosocial intervention group for older people with schizophrenia. *Behavioural and Cognitive Psychotherapy, 42*(2), 199-210.

90. Pence, B. W., Gaynes, B. N., Atashili, J., O’Donnell, J. K., Kats, D., Whetten, K., . . . Ndumbe, P. (2014). Feasibility, safety, acceptability, and preliminary efficacy of measurement-based care depression treatment for HIV patients in Bamenda, Cameroon. *AIDS and Behavior, 18*(6), 1142-1151. doi: 10.1007/s10461-014-0727-x

91. Borland, R., Li, L., Mortimer, K., McNeil, A., King, B., & O’Connor, R. J. (2011). The acceptability of nicotine containing products as alternatives to cigarettes: Findings from two pilotstudies. *Harm Reduction Journal, 8*, 1-7.

92. Mills, K. L., Ewer, P., Dore, G., Teesson, M., Baker, A., Kay-Lambkin, F., & Sannibale, C. (2014). The feasibility and acceptability of a brief intervention for clients of substance use services experiencing symptoms of post traumatic stress disorder. *Addictive Behaviors, 39*(6), 1094-1099.

93. Whicher, E. V., Utku, F., Schirmer, G., Exam, S., Davis, P., & Abou-Saleh, M. T. (2012). Pilot project to evaluate the effectiveness andacceptability of single-session brief counseling for the prevention of substance misuse in pregnant adolescents. *Addictive Disorders & Their Treatment, 11*(1), 49-49.

94. Kypri, K., Langey, J. D., Saunders, J. B., & Cashell-Smith, M. L. (2007). Assessment may conceal therapeutic benefit: findings from a randomized controlled trial for hazardous drinking. *Addiction, 102*, 62-70. doi: 10.1111/j.1360-0443.2006.01632.x

95. Bernstein, J. A., Bernstein, D., & Heeren, T. (2010). Mechanisms of change in control group drinking in clinical trials of brief alcohol intervention: Implications for bias toward the null. *Drug and Alcohol Review, 29*, 498-507. doi: 10.1111.j.1465-3362.2010.00174.x

96. Larsen, D., Attkisson, C., Hargreaves, W., & Nguyen, T. (1979). Assessment of client/patient satisfaction: Development of a general scale. *Evaluation and Program Planning, 2*, 197-207.

97. Fals-Stewart, W., O'Farrell, T. J., Frietas, T. T., McFarlin, S. K., & Rutigliano, P. (2000). The timeline followback reports of psychoactive substance use by drug-abusing patients: Psychometric properties. *Journal of Consulting and Clinical Psychology, 68*, 134-144.

98. Sobell, L. C., Brown, J., Leo, G. I., & Sobell, M. B. (1996 ). The reliability of the alcohol timeline followback when administered by telephone and by computer. *Drug and Alcohol Dependence, 42*, 49-54.

99. Sobell, L., Buchan, G., Cleland, P., Sobell, M., Federoff, I., & Leo, G. (1996). *The reliability of the Timeline Followback (TLFB) method as applied to drugs, cigarette, and cannabis use*. Paper presented at: 30th Meeting of the Association for the Advancement of Behavior Therapy. New York, New York.

100. Sobell, L. C., & Sobell, M. B. (1978). Validity of self-reports in three populations of alcoholics. *Journal of Consulting and Clinical Psychology, 46*(901-907).

101. Babor, T., Stephens, R., & Marlatt, G. (1987). Verbal report methods in clinical research on alcoholism: Response bias and its minimization *Journal of Studies on Alcohol, 48*, 410-424.

102. Sobell, M. B., Sobell, L. C., Klainer, F., Pavan, D., & Basian, E. (1986). The reliability of a timeline method for assessing normal drinker college students’ recent drinking history: Utility for alcohol research *Addictive Behaviors, 11*, 149-161.

103. McLellan, A. T., Alterman, A. I., Cacciola, J., Metzger, D., & O'Brien, C. P. (1992). A new measure of substance abuse treatment. initial studies of the treatment services review. *Journal of Nervous and Mental Disease, 180*, 101-110.

104. Straus, M. A., Hamby, S. L., McCoy, S. B., & Sugarman, D. B. (1996). The Revised Conflict Tactic Scales (CTS2). *Journal of Family Issues, 17*, 283-316.

105. Miller, W. (1999). *Enhancing motivation for change in substance abuse treatment*. ((SMA) 99-3354). Rockville, MD.

106. Sobell, L., Sobell, M., Toneatto, T., & Leo, G. (1993). What triggers the resolution of alcohol problems without treatment. *Alcohol, clinical, and experimental research, 17*(2), 217-224.

107. Sobell, L., Cunningham, J., Sobell, M., Agrawal, S., Gavin, D., Leo, G., & Singh, K. (1996). Fostering self-change among problem drinkers: a proactive community intervention. *Addictive Behaviors, 21*(6), 817-833.

108. Hesse, M. (2006). The Readiness Ruler as a measure of readiness to change poly-drug use in drug abusers. *Harm Reduction Journal, 3*(3).

109. LaBrie, J., Quinlan, T., Schiffman, J., & Earleywine, M. (2005). Performance of alcohol and safer sex change rulers compared with readiness to change questionnaires. *Psychology of addictive behaviors: Journal of the Society of Psychologists in Addictive Behaviors, 19*(1).

110. Sullivan, C., & Bybee, D. (1999). Reducing violence using community-based advocacy for women with abusive partners. *Journal of Consulting and Clinical Psychology, 67*, 43-53.

111. Johnson, J., & Zlotnick, C. (2008). A pilot study of group interpersonal psychotherapy for depression in substance-abusing female prisoners. *J Subst Abuse Treat, 34*(4), 371-377. doi: S0740-5472(07)00153-5 [pii]10.1016/j.jsat.2007.05.010

112. Zlotnick, C., Miller, I., Pearlstein, T., Howard, M., & Sweeney, P. (2006). A preventive intervention for pregnant women on public assistance at the risk for postpartum depression. *American Journal of Psychiatry, 163*(8), 1443-1445.

113. Zlotnick, C., Najavits, L. M., Rohsenow, D. J., & Johnson, D. M. . (2003). A cognitive-behavioral treatment for incarcerated women with substance abuse disorder and posttraumatic stress disorder: Finding from a pilot study *Journal of Substance Abuse Treatment 25*, 99-105.

114. Rounsaville, B. J., Carroll, K. M., & Onken, L. S. (2001). A state model of behavioral therapies research: Getting started and moving on from stage I. . *Clinical Psychology: Science and Practice, 8*(2), 133-142.

115. Ambrosius, W. (2009). *Developing an analysis plan*. Paper presented at the The Ninth Annual Summer Institute on Randomized Clinical Trials Involving Behavioral Interventions, Warrenton, VA.

116. Johnson, D., Zlotnick, C., & Perez, S. (2011). Cognitive behavioral treatment of PTSD in residents of battered women's shelters: results of a randomized clinical trial. *Journal of Consulting and Clinical Psychology, 79*(4), 542-551. doi: 10.1037/a0023822

117. Regan, K., Bartholomew, K., Kwong, M., Trinke, S., & Henderson, A. (2006). The relative severity of acts of physical violence in heterosexual relationships: An item response theory analysis. *Personal Relationships, 13*, 37-52.

118. U.S. Department of Health and Human Services. (2009). Research involving individuals with questionable capacity to consent: NIH points to consider.

119. Sullivan, C. M., & Cain, D. (2004). Ethical and safety considerations when obtaining information from or about battered women for research purposes. *Journal of Interpersonal Violence, 19*, 603-618.

120. Thompson, M., Basile, K., Hertz, M., & Sitterle, D. (2006). *Measuring intimate partner violence victimisation and perpetration. A compendium of assessment tools*. Atlanta, GA: National Center for Injury Prevention and Control.

121. Hegarty, K., Bush, R., & Sheahan, M. (2005). The composite abuse scale: Further development and assessment of reliability in two clinical settings. *Violence and Victims, 20*(5), 529-547.

122 McFarlane, J., & Parker, B. (1994). Preventing abuse during pregnancy: an assessment and intervention protocol. *American Journal of Maternal Child Nursing, 19*, 321-324.

123 McFarlane, J., Parker, B., Soeken, K., Silva, C., & Reel, S. (1998). Safety behaviors of abused women after an intervention during pregnancy. *Journal of Obstetrics and Gynecological Neonatal Nursing, 27*(1), 64-69.
